# New Perspectives on the Sustainable Employment of Chestnut Shells as Active Ingredient against Oral Mucositis: A First Screening

**DOI:** 10.3390/ijms232314956

**Published:** 2022-11-29

**Authors:** Ana Sofia Ferreira, Ana Margarida Silva, Diana Pinto, Manuela M. Moreira, Ricardo Ferraz, Jaroslava Švarc-Gajić, Paulo C. Costa, Cristina Delerue-Matos, Francisca Rodrigues

**Affiliations:** 1 REQUIMTE/LAQV—Instituto Superior de Engenharia do Porto, Rua Dr. António Bernardino de Almeida, 431, 4249-015 Porto, Portugal; 2 Ciências Químicas e Das Biomoléculas (CQB) e Centro de Investigação Em Saúde e Ambiente (CISA), Escola Superior de Saúde do Instituto Politécnico do Porto, 4400-330 Porto, Portugal; 3 Faculty of Technology, University of Novi Sad, Bulevar cara Lazara, 1, 21000 Novi Sad, Serbia; 4 REQUIMTE/UCIBIO—MedTech-Laboratory of Pharmaceutical Technology, Department of Drug Sciences, Faculty of Pharmacy, University of Porto, Rua de Jorge Viterbo Ferreira, 228, 4050-313 Porto, Portugal

**Keywords:** *Castanea sativa* shells, phenolic compounds, Subcritical Water Extraction (SWE), antimicrobial activity, oral mucositis

## Abstract

Oral mucositis (OM), a common side effect of oncological treatment, is an oral mucosal disorder characterized by painful ulcerations and increased risk of infection. The use of natural antioxidants to suppress the redox imbalance responsible for the OM condition has emerged as an interesting approach to prevent/treat OM. This study aims to explore the chestnut (*Castana sativa*) shells as potential active ingredient against OM. Therefore, chestnut shells were extracted at different temperatures (110–180 °C) by Subcritical Water Extraction (SWE), aiming to recover antioxidants. The extracts were also evaluated against microorganisms present in the oral cavity as well as on human oral cell lines (TR146 and HSC3). The highest phenolic content was obtained with the extraction temperature of 110 °C, exhibiting the best antioxidant/antiradical activities and scavenging efficiencies against HOCl (IC_50_ = 4.47 μg/mL) and ROO^•^ (0.73 μmol TE/mg DW). High concentrations of phenolic acids (e.g., gallic and protocatechuic acids) and flavanoids (catechin, epicatechin and rutin) characterized the phenolic profile. The antimicrobial activity against several oral microorganisms present in the oral cavity during OM, such as *Streptococcus*, *Staphylococcus*, *Enterococcus,* and *Escherichia*, was demonstrated. Finally, the effects on HSC3 and TR146 cell lines revealed that the extract prepared at 110 °C had the lowest IC_50_ (1325.03 and 468.15 µg/mL, respectively). This study highlights the potential effects of chestnut shells on OM.

## 1. Introduction

Oral mucositis (OM) is one of the most common side effects of antineoplastic treatments, namely chemo- and/or radiotherapy, and is defined as an acute inflammatory, ulcerative, and painful disorder that severely degrades the quality of life of patients [1,2,3]. Ulceration of buccal mucosa compromises the natural defensive barrier, allowing oral bacteria and inflammatory cytokines to enter underlying tissues and the systemic blood supply [2]. During the antineoplastic treatments, reactive oxygen species (ROS) are generated, leading to the development of oxidative stress, one of the principal phenomena in the basis of OM development [2,3]. An imbalance between the body’s synthesis of oxidants and antioxidant defenses of human body is known as oxidative stress, which may result in a wide range of diseases, including diabetes, cardiovascular or neurodegenerative diseases, and, at the last step, cancer [4,5]. Therefore, the oxidative stress pathway could be a therapeutic target against OM, particularly in what concerns natural antioxidants that have a strong potential to prevent and control the generation of free radicals and restore cellular homeostasis [6]. A recent analysis by our research team highlighted the benefits of using natural substances to treat this oral condition [1]. As the pharma-, nutra-, and cosmeceutical industries shift their focus on natural bioactive chemicals, natural product research is increasing due to their unique properties as source of therapeutic phytochemicals, commonly associated with efficacy, safety, and minimal side effects [7,8]. Among natural products, food by-products arise as a unique opportunity, considering sustainable principles and circular economy questions, such as low cost, abundance, and accessibility. Agro-industries generate large amounts of by-products in the form of shells, skins, flowers, leaves, and pulps, that may be used as raw resources to establish added-value products, a key strategy for a sustainable production [9,10]. This is the case of chestnut shells. European or sweet chestnut (*Castanea sativa*, Mill.) production has, as its primary purpose, the natural fruit consumption, or the production of frozen chestnut [11,12]. Portugal is one of the major European producers, with its main distribution coming from the northeast part of the country, covering 35,000 ha, with an estimated fruit production of 1800 kg/ha/year by 2022, expected to rise to about 3000 kg/ha/year in 2030 [11]. The inner and outer shells are generated by chestnut peeling processes, representing almost 10–15% of the whole chestnut weight [12,13]. Shells are mostly used as fuel and as source of tannins for wine aging, also being a major origin of fermentable sugars for the development of biofuels, while the extracts are known for their high polyphenol content (with emphasis on phenolic acids, flavonoids, and tannins) [12,13,14].

Extraction is the most vital phase for the recovery of bioactive compounds from natural matrices. A great challenge to start implementing eco-friendly and effective extraction methods, rather than the use of standard solvent extraction, has arisen in industry [15,16]. Greener alternative technologies, such as Subcritical Water Extraction (SWE), are being described as more ecological and efficient to recover valued compounds and produce high-quality extracts [17,18]. SWE presents a major advantage by using water as solvent, which is heated at a temperature range of 100–374 °C and a pressure state between 10–100 bar to maintain in its liquid form [19,20]. Under these parameters, water’s dielectric constant decreases, exhibiting a behavior like organic solvents and enabling a faster extraction, higher extraction yield, and efficient recovery of both polar and nonpolar compounds [19]. The short extraction time, energy savings, selectivity, discontinuation of organic solvents, as well as the low environmental impact and high cost-effectiveness ratio are positive aspects that should be considered [21,22]. Nevertheless, some phenolic compounds can be completely or partially degraded at high temperatures, producing new ones. Therefore, temperature is a key parameter in the SWE [19,21,23]. In 2019, our research team published the first study that evaluated the subcritical water extraction of chestnut shells, but the influence of temperature was not deeply explored, posing a challenge. Determining the ideal SWE temperature (110–180 °C) to extract bioactive compounds from *C. sativa* shells was the main aim of this study, to produce an optimal extract with promising effects against OM. While the *in vitro* effects on several buccal cell lines and the antibacterial activities against oral pathogens ensure the final OM effects, the phenolic profiles were evaluated to understand the specific compounds responsible for the antioxidant/antiradical activities. To the best of our knowledge, this is the first research that provides insights on how OM can be treated using a food by-product.

## 2. Results and Discussion

### 2.1. Extraction Yield

Extraction is a critical step in the phytochemical processing pathway to obtain the highest quantity of bioactive compounds from plant sources. To standardize plant products, it is crucial to use an appropriate extraction method [24,25]. Furthermore, for upscaling reasons, it is crucial to select an appropriate extraction technique and optimize various parameters, such as temperature, sample-to-solvent ratio, pH, and extraction period [22]. Various extraction techniques such as maceration, infusion and Soxhlet extraction, and alternative and greener methods, such as microwave-assisted extraction (MAE), ultrasound-assisted extraction (UAE), supercritical fluid extraction (SFE), and SWE, are available in the market [18,24,26,27,28]. Green extraction techniques are faster and more environmentally friendly in terms of solvent and energy use, leading to a continuously increasing application in recent years. In some circumstances, the green extraction yield is considerably higher than that of the conventional approaches [26,28,29,30]. Table 1 summarizes the extraction yields of the different extracts of chestnut shells obtained at different temperatures (110 °C, 120 °C, 140 °C, 160 °C, and 180 °C).

As it is possible to observe, no significant extraction differences were observed between the extract’s yields, with the extract prepared at 180 °C achieving the lowest value (20.29%), oppositely to the extract obtained at 120 °C (21.00%). Cacciola et al. (2019) evaluated three different methods (conventional liquid extraction, UAE, and MAE) using water as a solvent to recover bioactive compounds from chestnut shells. According to the authors, the extraction yields obtained for conventional liquid extraction, UAE, and MAE were, respectively, 5.2%, 2.2%, and 3.8% [31], at least four times lower than those shown in Table 1. In another study, Fernández-Agulló et al. (2014) reported higher extraction yields for chestnut shells extracted using 50% of organic solvents, namely methanol (12.09%) and ethanol (13.27%), when compared to conventional extraction at 75 °C with water (8.72%) and MAE (10.75%, 10.38% and 5.64%, respectively, for methanol, ethanol, and water) [32]. The authors demonstrated the influence of temperature on the extraction efficiency, as the extraction yields increased with the augmented extraction temperatures tested [32]. In addition, Pinto et al. (2021) determined the efficiency of MAE in extracting *C. sativa* shells and reported extraction yields ranging from 6.52% (25% aqueous extract) to 11.13% (100% ethanol) [33]. The same research group tested different SWE times and stated yields between 6.70 and 9.19% [19].

The influence of sample-to-solvent ratio and mass transport enhancement on chestnut shells extraction was also evaluated in Microwave-Assisted Subcritical Water Extraction (MASWE) using a range of temperatures between 100 °C and 220 °C [34]. According to the authors, it was evidenced an increase of the extract production proportional to the increase of temperature, but minor deviations were observed for the sample-to-solvent ration (1:20–1:30), with exception of the 150 °C extract (322.37 mg/g DW and 416.56 mg/g DW, respectively) [34].

The SWE extraction yields reported in Table 1 were consistent with those determined by Wang et al. (2014) for citrus peel [35], Silva et al. (2022) for kiwiberry leaves [36], and Rodrigues et al. (2019) for papaya seed extraction [37].

### 2.2. Extraction Yield

Table 1 summarizes the TPC, and antioxidant/antiradical activities of SWE extracts obtained at different temperatures. As shown, the TPC ranged between 126.19 and 239.53 mg GAE/g DW. The highest results were obtained for the extracts prepared at 120 °C (201.75 mg GAE/g DW) and 110 °C (239.53 mg GAE/g DW), and significant differences were noted between the extracts obtained at 120 °C and 140 °C (*p* = 0.008). Thermal degradation is probably responsible for the decrease in polyphenols content at higher temperatures. Nonetheless, this phenomenon alone cannot explain polyphenol behavior, as in conventional extraction methods, and the polyphenols breakdown can also occur at 80 °C. In fact, the increase in TPC observed at high SWE temperatures could also be attributed to the disruption of lignin–phenolic acid linkages or to the breakdown of lignin itself, resulting in large amounts of phenolic acids [23].

Pinto et al. (2021) assessed the phenolic profile, bioactivity, and cell viability of *C. sativa* shells extracted by MAE using different solvents (water and ethanol). The research team concluded that the highest TPC was achieved by the aqueous extract (173.89 mg GAE/g DW) [33]. The same authors tested this matrix by SWE extraction using response surface methodology (RSM) and reported higher TPC values for the extract prepared at 220 °C (417.30 mg GAE/g DW); however, the phenolic profile was different from that obtained in the present study. According to the authors, the response surface 3D plots of the TPC showed that the temperature of 220 °C was the optimal temperature and the phenolic content at 80 °C is higher than that at 150 °C, probably due to the degradation of tannin [19].

IC_50_ values for the DPPH assay ranged from 426.88 g/mL (110 °C) to 583.47 μg/mL (180 °C). Statistical analysis demonstrated significant differences between the extracts (*p* = 0.002), which aligns with the TPC results. At lower temperatures the extracts presented higher phenolic content, resulting in higher antioxidant powers.

Regarding the ABTS assay, the IC_50_ results varied between 148.68 μg/mL and 256.59 μg/mL for the extracts obtained at 110 °C and 180 °C, respectively. Significant differences were noted between the extracts obtained at 110 °C, 120 °C, and 140 °C (*p* < 0.016), as well as between the extracts obtained at 120 °C, 160 °C, and 180 °C (*p* < 0.05).

According to Table 1, the extract’s ability to decrease ferric ions increased with temperature was 180 °C < 160 °C < 140 °C < 120 °C < 110 °C. The extract processed at 110 °C had the highest value (4240.38 μmol FSE/g DW), while the extract produced at 180 °C had the lowest activity (2464.32 μmol FSE/g DW), an almost two-fold difference. Significant differences (*p* = 0.031) were observed between the extracts obtained at 110 °C and 160 °C.

Fernández-Agulló et al. (2014) also used DPPH and FRAP assays to evaluate the antiradical and antioxidant properties of chestnut shells and found that the aqueous extract (75 °C) obtained by conventional extraction produced the best results (IC_50_ = 0.031 mg/mL for DPPH; IC_50_ = 0.284 mg/mL for ABTS). The same extract obtained the highest FRAP (56.23 g GAE/100 g extract), in the accordance with TPC results [32]. In the chestnut shells SWE study, the extract prepared at 220 °C was assessed through DPPH, FRAP, and ABTS assays and showed good results, respectively, 901.16 mg AAE/g DW, 7994.26 mg FSE/g DW and 815.66 mg TE/g DW [19]. Regarding the UAE extraction of chestnut shells, the extract (70 °C/40 min) also had good antiradical and antioxidant results in the DPPH (IC_50_ = 44.1 µg/mL), ABTS (IC_50_ = 65.4 µg/mL) and FRAP (IC_50_ = 32.0 µg/mL) assays [38]. Analyzing the results from Table 1 with the authors mentioned above, it is evident that the extraction conditions are crucial. When the plant matrix was the same, the different extraction methods used were subjected to several variations. A detailed understanding of the bioactive composition, particularly the phenolic profile, is, therefore, essential, since the spectrophotometric assays have limitations such as the sensitivity to pH, reaction time, and presence of interferents (e.g., sugars and amino acids) [39].

### 2.3. Phenolic Profile Identification and Quantification by HPLC-PDA

The phenolic profiles of the *C. sativa* shells extracts prepared using SWE are detailed in Table 2.

As shown in Table 2, health-promoting compounds were grouped into seven different classes. Overall, 25 phenolic compounds were identified and quantified, with phenolic acids being the main class of compounds. The extract obtained at 110 °C presented the highest amount of phenolic acids (20.20 mg phenolic acids/g DW), though higher temperatures led to a significant decrease, such as the extract prepared at 160 °C (7.07 mg phenolic acids/g DW). This may be due to the diminution of the dielectric constant and polarity of water as temperature increases, which leads to the dissolution of non-polar compounds such as polyphenols [40]. However, high temperatures have a negative effect on polyphenols above 120 °C, resulting in the breakdown of thermally unstable polyphenols [41].

As it is possible to observe, the major phenolic acid present in all extracts is gallic acid, particularly at 110 °C, 120 °C and 140 °C (5.99, 5.08 and 4.64 mg/g DW, respectively). This compound is extremely important for human health, and several studies have reported its biological and pharmacological activities, including anti-inflammatory, antioxidant, anticancer, and antimicrobial activities [42]. Moreover, the presence of protocatechuic acid may indicate the thermal degradation of catechin [19]. Flavonoids were also identified in considerable amounts, corresponding to more than 30% of the total phenolic content. Particularly, catechin ranged from 3.65 to 4.10 mg/g DW for the extracts obtained at 120 °C and 110 °C, respectively. The epicatechin amounts in the extracts obtained at 110 °C and 120 °C were, respectively, 1.68 and 1.14 mg/g DW, while for rutin, the quantities were 1.04 (110 °C) and 0.64 mg/g DW (120 °C). However, for most of the compounds mentioned, the results indicate a high temperature sensitivity that should be considered.

Oxidative stress is the main factor in the basis development of OM, which induces to the activation of the apoptotic process, leading to the clinical manifestation of mucosal damage. Pro-inflammatory cytokines are in a positive feedback mechanism, conducting to ulceration, which compromises the mucosal integrity, allowing several microorganisms to penetrate tissues and increasing the sepsis risk [2,43]. The five phenolic compounds mentioned above were found at higher concentrations in all the extracts. Because of their capacity to control crucial cellular enzyme activities that lead to anti-inflammatory, antioxidant, antimicrobial, and anticancer activities, all of them have been associated with a wide range of beneficial properties and are expected to be potential treatments and prevention options for OM [44].

Previous studies have reported discrepancies in the phenolic composition of chestnut shells extracts, which can be explained by the use of diverse extraction techniques and also varied extraction settings. For example, Vásquez et al. (2012) analyzed chestnut shells extracted by organic solvents using conventional methods. The authors reported 11 compounds, with catechin/epicatechin and gallocatechin/epigallocatechin being the principal ones, while hydrolysable tannins, such as galloyl-glucoses and ellagic acid, were also present and identified [45]. Gallic acid esters of glucose were particularly present in chestnut shells extracted with water at 75 °C (conventional extraction) as well as gallocatechin and catechin [32]. In the chestnut shells extract prepared by UAE, ellagic acid (40.4 µg/mg DW), caffeic acid derivative (15.4 µg/mg DW) and epigallocatechin (15.3 µg/mg DW) were identified in high quantities [38], while by MAE the gallic acid and protocatechuic acid were also detected (117.58 and 16.8 mg/g DW, respectively) [31].

### 2.4. ROS Scavenging Capacity

A major global issue is the development of disorders related to oxidative stress caused by an imbalance between reactive species and antioxidants commonly present in the human body, being a key factor for the OM pathogenesis. However, its mechanism remains unclear. Nonetheless, it is known that chemo and/or radiotherapy triggers an increase in ROS generation [1]. Reactive species, such as hypochlorous acid (HOCl), superoxide (O_2_^•−^), peroxyl radical (ROO^•^), and peroxynitrite (ONOO^-^), when produced in excess in living cells are responsible, directly, or indirectly, for a number of diseases, including OM and, at last stage, oral cancer [46,47]. One of the most significant classes of natural antioxidants that can prevent or reduce the harm caused by oxidative stress is the polyphenol family, which has a positive effect on OM [43]. Table 3 shows the ROS scavenging activity results.

The extract prepared at 140 °C (IC_50_ = 31.14 μg/mL) showed the highest activity in terms of O_2_^•−^ scavenging capacity, whereas the extract prepared at 180 °C showed the lowest activity (IC_50_ = 73.18 μg/mL). Gallic acid was used as a positive control for this test, and it produced the best results (IC_50_ = 23.82 μg/mL). No significant differences (*p* = 0.123) were observed between the extracts. However, HOCl was effectively scavenged by all extracts. While the extract obtained at 160 °C had the lowest free radical scavenging capacity (IC_50_ = 22.85 °C), the extract prepared at 110 °C had the best effectiveness (IC_50_ = 4.47 °C). Significant differences were noted between extracts obtained at 110 °C and 140 °C (*p* = 0.026), and 160 °C and 180 °C (*p* = 0.027). Regarding the ROO^•^ quenching, the efficacy increased in the following order: gallic acid >110 °C > 140 °C > 120 °C >160 °C >180 °C. No significant difference (*p* = 0.379) was observed between extracts.

Comparing with other studies, the SWE extracts prepared in the present study showed lower ROS scavenging activity. Lameirão et al. (2021) related that the chestnut shells extract obtained by UAE (at 70 °C during 40 min) achieved an IC_50_ of 14.1 µg/mL, 0.7 µg/mL and 0.3 µg/mL regarding O_2_^•−^, HOCl and ROO^•^, respectively [38]. Likewise, the optimal SWE extract (obtained at 220 °C) of chestnut shells prepared by Pinto et al. (2021) exhibited high scavenging activity regarding HOCl (IC_50_ = 0.79 µg/mL) and O_2_^•−^ (IC_50_ = 12.92 µg/mL). However, as summarized in Table 3, the extracts in the present study had a better ability to quench ROO^•^, as Pinto et al. reported an Ssample/STrolox = 0.084 for the optimal extract [19].

Regarding RNS, the peroxynitrite (ONOO^-^) scavenging activity was evaluated in the absence and in presence of hydrogen carbonate (HCO_3_^−^) to mimic the in vivo environment. Although the results presented in Table 3 do not attest to a temperature-dependent correlation, a significant ability of the extracts to act as effective reducers of peroxynitrite-mediated damage can be observed. The best IC_50_ calculated was 1.41 µg/mL (for the extract prepared at 120 °C) and 1.89 µg/mL (for the extract obtained at 160 °C) in the presence and absence of HCO_3_^−^, respectively. However, the gallic acid achieved best results (IC_50_ = 0.30 and 0.05 µg/mL, respectively, in presence and absence of HCO_3_^−^). To the best of our knowledge, this is the first peroxynitrite assay performed on chestnut shells extracts. The phenolic profile, and thus the potential for compounds to degrade, as previously mentioned, could play a role in the observed discrepancies. No significant differences between the extracts were observed in the presence (*p* = 0.285) or absence (*p* = 0.357) of HCO_3_^−^. However, the radical scavenging capacity calculations, particularly for HOCl and ROO^•^, are generally consistent with the spectrophotometric results (Table 1), indicating that the extract prepared at 180 °C had the lowest potential, while the extract obtained at 110 °C had the most promising characteristics for OM treatment.

### 2.5. Antimicrobial Activity

The oral cavity contains complex microbiota compromising more than 700 different bacterial species. The oral cavity is an ideal environment for microbial growth due to its pH (6.5–7.5), temperature (37 °C), soft tissues (palate, tongue, and buccal mucosa), and hard tissues (teeth) [48]. The oral microbial population is generally balanced however, pathologies may appear when the ecological balance is disturbed. Chemotherapy-induced myelosuppression and radiation-induced xerostomia are known to cause microbiome dysbiosis, which compromise the saliva flow, a major barrier against potential pathogens [49]. Owing to its ability to affect the innate immune response, which is an activator of the mucositis pathway, the oral microbiota can aggravate or sustain oral mucositis [50]. Examples of the main species of bacteria commonly isolated from the oral cavity are *Porphyromonas*, *Lactobacillus*, *Veillonella*, *Actinobacillus*, *Streptococcus*, *Staphylococcus, Enterococcus*, and *Escherichia* [51,52].

The antimicrobial capacity of SWE *C. sativa* shells extracts was analyzed against several Gram-negative and Gram-positive bacteria, the majority of which are present in the oral cavity in case of OM [52,53]. MIC and MBC were determined for each microorganism at different extract concentrations (2–64 mg/mL) using a microplate dilution assay. Table 4 summarizes the results for all strains.

As can be observed, the chestnut shells extracts obtained at different temperatures exhibited diverse effects on the growth of microorganisms, which is probably due to the phenolic content of the extracts.

The extract prepared at 110 °C did not present MIC and MBC values for *S. aureus* (Gram-positive) at the concentrations tested. However, the extract showed a significant antimicrobial activity against two drug-resistant bacteria, *S. aureus* MRSA and *E. coli* CTX M2 (MIC of 4 mg/mL and MBC of 32 mg/mL in *S. aureus* MRSA). This extract also exhibited MIC in *E. coli* ATCC 8739, *E. coli* ATCC 25922, and *E. faecalis* (8, 4, and 64 mg/mL, respectively) and MBC of 64 mg/mL in *E. coli* ATCC 25922. *E. coli* CTX M2 was resistant to the extract obtained at 180 °C. Compared to the 110 °C extract, the extract prepared at 180 °C presented higher MIC values for the two non-resistant strains of *E. coli* (8 mg/mL), but a lower MIC for *E. faecalis* (16 mg/mL). At this temperature, the extract exhibited MBC values for all strains that had growth inhibition. As shown in Table 4, the extract prepared at 160 °C showed better antimicrobial activity, as it showed lower MIC values for most of the strains tested and displayed MBC values for all the strains, except for the drug-resistant *E. coli* CTX M2.

Phenolic compounds may affect bacterial cells by damaging their cellular membranes, binding to the cell wall, and leading to enzyme inactivation and DNA damage [54]. Flavanols (e.g., catechin and epicatechin) are known to inhibit the in vitro growth of several strains, including *S. mutans* and *E. coli* [55]. Flavonols (e.g., rutin) also exhibit a significant activity against *S. aureus* (Gram-positive), possibly through an aggregation mechanism. Nevertheless, phenolic acids, such as gallic acid, also showed antibacterial activity against *S. aureus* and *E. coli*. Although these compounds present weaker efficiency in bacterial growth inhibition when compared to flavonoids, they still prove to be more efficient than standard antibiotics, such as gentamicin and streptomycin [55,56]. Kang et al. (2008), studied the antimicrobial effects of gallic acid, one of the main compounds present in *Rhus chinensis* L., and reported MIC values of 8 mg/mL for *S. mutans*. The authors also reported in vitro inhibition of *S. mutans* biofilms, demonstrating the antimicrobial activity of gallic acid [57]. Likewise, Passos et al. (2021) reported the antimicrobial and anti-adherence activity of gallic acid, an isolated compound of the ethanolic extract of *L. ferrea*, which exhibited significant antimicrobial activity and inhibition of adhesion of *S. mutans* biofilms [58].

Previous research has demonstrated a correlation between phenolic compounds and the antimicrobial activity, as the quantity of phenolic compounds has a proportional inhibitory effect on the microorganism growth [59]. Though, the present results do not demonstrate a correlation as the extract prepared at 110 °C has a higher amount of phenolic compounds and the extract obtained at 160 °C showed better antimicrobial capacity. One of the possible explanations for such data is the antagonistic effect of the combination of polyphenolic compounds present in the SWE extracts, which may decrease the antimicrobial ability.

### 2.6. Cells Viability Assays

**Figure 1:**
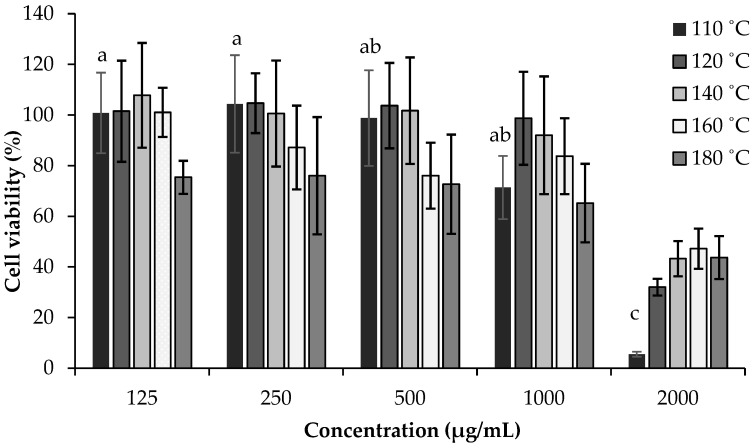
Effects of *C. sativa* shells SWE extracts on the viability of HSC3 cells at a range of concentrations of 125–2000 µg/mL, measured by MTT assay, at 24 h. Values are expressed as mean ± standard deviation (*n* = 3). Different letters indicate significant differences between concentrations of the same sample (*p* < 0.005) according to Tukey’s HSD test.

Considering the potential use of SWE *C. sativa* extracts in OM treatment, it is particularly important to assess their effects in in vitro models. In this study, SWE *C. sativa* shells extracts were evaluated on HSC3 and TR146 cell lines by MTT assay. Both cell lines mimic the normal human buccal epithelium and are tumorigenic, making them suitable as oral mucosal models for testing medication absorption. In particular, the TR146 cell line was established from a neck metastasis of a buccal carcinomic tumor, and the HSC3 cell line was derived from tumors of metastatic lymph nodes originating from tongue squamous cell carcinoma [60,61,62]. Figure 1 summarizes the obtained results for both cell lines using various extract concentrations (125–2000 μg/mL), while Table 5 presents the IC_50_ values.

**Figure 2:**
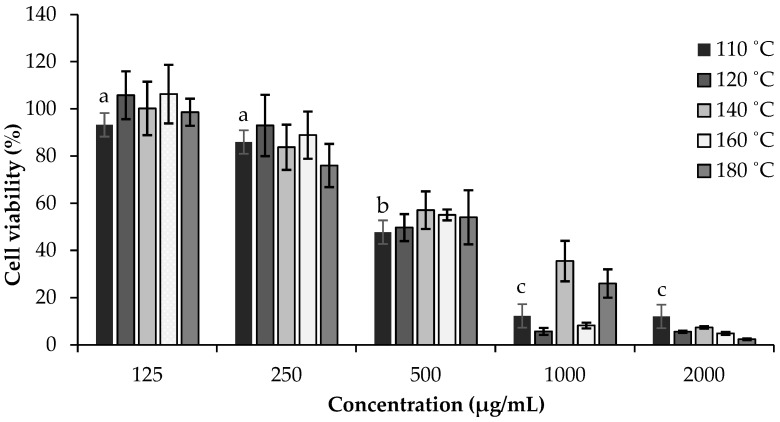
Effects of *C. sativa* shells SWE extracts on the viability of TR146 cells at concentrations range of 125–2000 µg/mL, measured by MTT assay, at 24 h. Values are expressed as mean ± standard deviation (*n* = 3). Different letters indicate significant differences between concentrations of the same sample (*p* < 0.005), according to Tukey’s HSD test.

Regarding the HSC3 results, the extracts obtained at 160 °C and 180 °C presented viabilities of 87.16% and 76.03%, respectively, after exposure with the low concentrations tested (250 µg/mL). The extracts obtained at 110 °C, 120 °C, and 140 °C showed viability reductions at concentrations of 500, 1000, and 2000 µg/mL, respectively. Significant differences were noted for the different concentrations of the extracts obtained at 110 °C (500–2000 µg/mL; *p* = 0.044) and 180 °C (125–250 µg/mL and 125–2000 µg/mL; *p* < 0.03). These results might be explained by the fact that the extract prepared at 110 °C contained the highest concentration of phenolic acids, which was in accordance with Table 1. As shown in Figure 2, except for the extract prepared at 110 °C, none of the extracts obtained at different temperatures resulted in a reduction in the viability of TR146 cells at a concentration of 125 µg/mL. However, with increasing concentrations, all extracts significantly decreased cellular viability, leading to viability values below 57% for all extracts at 500 µg/mL. Significant differences were observed for the extract prepared at 140 °C (125–250 µg/mL; *p* = 0.022) and 180 °C (25–1000 µg/mL; *p* = 0.016). These results proved that TR146 cells were the most sensitive to the SWE chestnut shells extracts (Table 5). Moreover, it can be concluded that the extracts exhibited antiproliferative effects for both cell lines, with the extract prepared at 110 °C achieving the lowest IC_50_ value in TR146 (468.15 µg/mL) and in HSC3 (468.15 µg/mL). This may be due to the higher content of phenolic compounds, as shown in Table 1.

Lambert et al. reported that polyphenols can inhibit different phases of carcinogenesis, including initiation, promotion, and progression of tumor cells, demonstrating anticancer properties [63]. In addition, polyphenols have been reported to prevent oral cancer, as they come into direct contact with tissues prior to being absorbed, suppressing the proliferation of oral cancer cells on the surface of epithelial cells [64]. Catechins, for example, have been demonstrated the capacity to decrease the activity of oral squamous cell carcinoma (OSCC) in OC2 cells by inhibiting the synthesis of matrix metalloproteinases (MMPs). Hence, invasion and migration of cancer cells can be regulated [65,66]. To the best of our knowledge, this is the first study that assessed the effects of SWE chestnut shells extract on oral model cell lines. This highlights its potential as an ingredient against OM condition.

## 3. Materials and Methods

### 3.1. Chemicals

α,α′-azodiisobutyramidine dihydrochloride (AAPH), β-nicotinamide adenine dinucleotide (NADH), dihydrorhodamine 123 (DHR), 2,2-diphenyl-1-picryl-hydrazyl (DPPH), sodium hypochlorite solution with 4% available chlorine, phenazine methosulphate (PMS), nitroblue tetrazolium chloride (NBT), fluorescein sodium salt and Trolox were obtained from Sigma-Aldrich (Steinheim, Germany). Catechin, gallic acid, Folin–Ciocalteu’s reagent and Triton X-100 were purchased from Sigma Chemical Co. (St. Louis, MO, USA).

For microbial culture, Mueller Hinton Broth (MHB) medium was purchase to Liofilmchem (Teramo, Italy), Tryptic Soy Broth (TSB), and Tryptic Soy Agar (TSA) medium was obtained from Biolife (Milan, Italy). HPLC solvents were delivered by Sigma-Aldrich (Milan, Italy). Individual’s standards of phenolic compounds used for the identification and quantification in extracts were purchased from Sigma-Aldrich (Steiheim, Germany). HPLC solvents were delivered by Sigma-Aldrich (Milan, Italy). Individual’s standards of phenolic compounds, namely gallic acid (≥99%), protocatechuic acid (99.63%), neochlorogenic acid (≥98%), caftaric acid (≥97%), chlorogenic acid (>95%), 4-*O*-caffeyolquinic acid (≥98%), vanillic acid (≥97%), caffeic acid (≥98%), syringic acid (≥98%), *p*-coumaric acid (≥98%), *trans*-ferulic acid (≥99%), sinapic acid (≥99%), 3,5-di-*O*-caffeyolquinic acid (≥95%), ellagic acid (≥95%), 4,5-di-*O*-caffeyolquinic acid (≥90%), (+)-catechin (≥98%), (-)-epicatechin (≥90%), naringin (≥95%), quecetin-3-*O*-galactoside (≥97%), quercetin-3-*O*-glucopyranoside (≥99%), rutin (≥94%), phloridzin (99%), *trans*-polydatin (≥98%), resveratrol (≥99%) and caffeine (≥95%), used for the identification and quantification in extracts were purchased from Sigma-Aldrich (Steiheim, Germany). Dulbecco’s Modified Eagle Medium (DMEM), penicillin, trypsin-EDTA and streptomycin were provided by Invitrogen Corporation (Life Technologies, S.A., Madrid, Spain). Dimethyl sulfoxide (DMSO) was purchased by AppliChem (Darmstadt, Germany).

TR146 and HSC3 cell lines were obtained from the American Type Culture Collection (ATCC, Manassas, VA, USA). The following reference bacterial strains were obtained from the American Culture Collection (ATCC): *Escherichia coli* ATCC 25922, *E. coli* ATCC 8739, and *Staphylococcus aureus* ATCC 25913. All other strains, including resistant bacteria, were clinical isolates: *Enterococcus faecalis* and the resistant bacteria, *Escherichia coli CTX M2* and *Staphylococcus aureus MRSA*.

### 3.2. Sample

*Castanea sativa* shells were generously provided by Sortegel, located in Sortes, Bragança, Portugal. Dehydration of shells occurred at 41 °C for 24 h (Excalibur Food Dehydrator, Pennsylvania, USA) and then were pulverized in a miller to 1 mm particles (Ultra Centrifugal Mill ZM 200, Retsch, Germany). Prior to extraction, samples were carefully blended and kept at room temperature (20 °C), in the dark.

### 3.3. Subcritical Water Extraction (SWE) of C. sativa Shells

Extraction of *C. sativa* shells was performed in a homemade subcritical batch-type extractor of 1.7 L [67]. For it, a built-in valve and 99.99% nitrogen (Messer) pressurization were used. The samples were then extracted for 30 min at various temperatures between 110 °C and 180 °C using 20 bars of pressure and a 1:30 sample-to-solvent ratio. The vibrating platform (3 Hz) housing the extraction vessel agitated the sample/water mixture during the extraction. The extraction vessel was then cooled using a flow-through water bath (20 ± 2 °C), and the pressure was released by opening the valve. Filtration was used to separate the extracts, and the solutions were subsequently lyophilized (Telstar, model Cryodos −80 °C, Spain) and stored at room temperature (20 °C) until further analysis. The ratio between the total weight of the lyophilized extract and the total weight of the extract obtained following SWE was used to calculate the extraction yield as follows:

$$\mathrm{Extraction}\;\mathrm{yield}\;\left(\%\right)=\frac{\mathrm{weight}\;\mathrm{of}\;\mathrm{lyophilized}\;\mathrm{extract}}{\mathrm{weight}\;\mathrm{of}\;\mathrm{liquid}\;\mathrm{extract}}\times 100$$

### 3.4. Determination of Total Phenolic Content

The Folin–Ciocalteu method was used to measure the total phenolic content (TPC) using spectrophotometry, with minor changes [19]. A calibration curve (linearity range = 5–100 µg/mL; *R*^2^ > 0.996) was created using gallic acid solution. Results were expressed as mg of Gallic Acid Equivalents (GAE) per g of dry weight (mg GAE/g DW).

### 3.5. Antioxidant and Antiradical Activities

#### 3.5.1. Ferric Reducing Antioxidant Power (FRAP) Assay

FRAP assay was carried out as described by Benzie and Strain with a few minor changes [36]. A calibration curve (linearity range: 25–500 µM; *R*^2^ > 0.993) was established using a standard ferrous sulfate (FeSO_4_ ·7H_2_O) solution at a concentration of 1 mM. Results were expressed in µmol of ferrous sulfate equivalents (FSE) per gram of DW (µmol FSE/g DW).

#### 3.5.2. 2,2-Diphenyl-1-picrylhydrazyl (DPPH) Free Radical Scavenging Assay

DPPH free radical scavenging assay was performed according to the method described by Pinto et al. [68]. Trolox was used as reference for the calibration curve (linearity range: 5–125 µg/mL; *R*^2^ > 0.989). Results were presented as the radical scavenging activity’s half-maximal inhibitory concentration (IC_50_, µg/mL).

#### 3.5.3. 2,2´-Azino-bis-3-ethylbenzothiazoline-6-sulfonic Acid (ABTS) Radical Scavenging Activity Assay

Extracts were tested for ABTS radical scavenging activity according to Re et al. [69], with slight adjustments. The calibration curve standard (linearity range: 5–100 µg/mL; R^2^ > 0.998) was ascorbic acid and the results were expressed as IC_50_ (µg/mL).

### 3.6. HPLC-PDA Analysis

HPLC was used to identify and quantify polyphenols using photodiode array (PDA) detection [70]. Separation was performed at 25 °C using a Gemini C18 column (250 mm 4.6 mm, 5 m, Phenomenex, Alcobendas, Spain). Results on DW (mg/g DW) were represented as mg of each phenolic component per gram of extract. The calibration data used for the quantification of individual phenolic compounds in the chestnut shells extracts are presented in Table S1.

### 3.7. Reactive Oxygen Species and Reactive Nitrogen Species Scavenging Capacity

#### 3.7.1. Superoxide Radical Scavenging Assay

Superoxide radical (O_2_^•−^) scavenging assay was performed as described by Gomes et al. [71]. Results were expressed as IC_50_ of the NBT reduction to diformazan.

#### 3.7.2. Hypochlorous Acid Scavenging Assay

Hypochlorous acid (HOCl) scavenging assay was performed accordingly to Gomes et al. [71]. Gallic acid was used as a positive control. The results were presented as an inhibition of the HOCl-induced oxidation of DHR, measured in IC_50_.

#### 3.7.3. Peroxyl Radical Scavenging Assay

Peroxyl radical (ROO^•^) assay was carried out according to the method described by Ou et al. [72]. Trolox was used as standard control. The ratio of the slopes obtained for the extracts or positive controls to the slope of Trolox (Sample/STrolox), which represents the ROO^•^ induced oxidation of fluorescein, was used to express the results.

#### 3.7.4. Peroxynitrite Scavenging Assay

The effects of the studied extracts on the of inhibition of ONOO^-^ induced oxidation of non-fluorescent DHR to fluorescent rhodamine 123 were assessed by peroxynitrite (ONOO^-^) scavenging activity. ONOO^-^ was synthesized as described by Whiteman et al. [73]. To simulate physiological CO_2_ values, parallel tests were run in the presence of 187.5 mM NaHCO_3_, and the results were represented as IC_50_.

### 3.8. Antimicrobial Activity

#### 3.8.1. Minimum Inhibitory Concentration (MIC) and Minimum Bactericidal Concentration (MBC)

MIC values were determined by the broth microdilution method using a 96-well plate with MHB medium with minor modifications [74]. Microorganisms were exposed to extracts (0.05 mL of dilutions + 0.05 mL of MHB medium) in the following concentration range: 2–64 mg/mL. The extracts were dissolved in deionized water and the results were compared with those bacteria that had grown in MHB in the presence of water as a control. The MIC of each extract was determined as the lowest concentration that showed no turbidity after 24 h of incubation at 37 °C. All the 96-well plates and petri dish plates were sealed.

#### 3.8.2. Growth Rate (GR) Studies

The GR parameter was determined using the broth microdilution method in a 96-well plate using TSB. All different strains of bacteria were exposed to extracts concentrations superior, equal, or inferior to the determined MIC, performing 1 h interval readings of the optical density (OD) at 625 nm, 37 °C, for 24 h [74]. Growth rate was determined by fitting a linear function to an exponential (log) phase curve.

#### 3.8.3. Minimum Bactericidal Concentration (MBC) Determination

MBC assays were evaluated by the broth dilution method, where MBC was established as the lowest extract concentration that inhibited 99.9% of the bacterial inocula after 24 h of incubation at 37 °C. Briefly, 5 µL was taken from the well obtained from the MIC experiment from each extract and its dilutions and added to a petri dish plate with TSA medium. All the petri dish plates were sealed. The plate was divided into six equal areas, and for all extracts, the area of the lower dilution with no visible colonies was determined as MBC [75].

### 3.9. Cell Viability Assay

A cell viability assay was performed using two human oral cancer cell lines, namely TR146 and HSC3. Passages 28 and 6 were used for TR146 and HSC3, respectively. The cells were cultured in a CellCulture^®^ CO_2_ Incubator (ESCO GB Ltd., Barnsley, UK) for 24 h at 37 °C and exposed to various extract concentrations (125–2000 g/mL). This assay was performed according to Pinto et al. [19]. Extracts were dissolved in cell culture medium or absent and then incubated (2.5 × 10^4^ cells/mL). MTT reagent was added, and the cells were incubated for 3 h at 37 °C, the number of viable cells was estimated, and the crystals were solubilized using DMSO. The positive and negative control were DMEM and 1% (*w/v*) Triton X-100. The background absorbance (630 nm) was subtracted at the 590 nm reading. Cell viability results were expressed as percentages.

### 3.10. Statistical Analysis

Results are expressed as mean ± standard deviation (*n* = 3). IBM SPSS Statistics 27.0 software (SPSS Inc., Chicago, IL, USA) was used to conduct statistical analysis. Tukey’s HSD test was used for post hoc comparisons of the means, and one-way ANOVA was used to analyze sample differences. *p* values lower than 0.05 (*p* < 0.05) were recognized as significant.

## 4. Conclusions

The scientific data presented in this study support the valorization of *C. sativa* shells extracts generated by SWE, an innovative and environmentally safe extraction method that may be applied as a therapeutic agent for the treatment of OM. The extract that produced the best results in terms of antioxidant and antiradical activities as well as the ability to scavenge free radicals was that produced at 110 °C, which was the optimal temperature for extraction. This extract also had a higher variety of polyphenols (25 phenolic compounds), with a particular focus on gallic acid, protocatechuic acid, catechin, epicatechin, caffeine, and rutin. Furthermore, antimicrobial activity assays demonstrated that, in general, all extracts significantly inhibited bacterial growth against strains commonly found in OM patients. The chestnut shells extracts induced cytotoxic effects in HSC3 and TR146 cells, with a particular focus on TR146 cell line, and the extract obtained at 110 °C exhibited the lowest IC_50_ values for both cell lines. All the biological properties exhibited by the SWE *C. sativa* shells extract, particularly the extract obtained at 110 °C, may target the oxidative stress caused by anticancer treatments through the antioxidant activity and radical species scavenging capacity and, consequently, the inflammatory state. In addition, in advanced conditions (ulceration), it can manage bacterial proliferation and exert antiproliferative effects in head and neck squamous cell carcinoma. More research is required to reach a conclusion on the effects on the oral mucosa, particularly on the absorption of phenolic compounds via the buccal route. These may include in vitro oral epithelial permeation assays and oxidative stress experiments in cell lines. Furthermore, the sustainable use of chestnut shells will improve the applicability of food by-products, improve their economic value, and contribute to environmental sustainability.

## Figures and Tables

**Table 1:** Extraction yields, total phenolic content (TPC), and antioxidant/antiradical activities of *C. sativa* extracts prepared by SWE were evaluated by DPPH, ABTS, and FRAP assays. Results are expressed as mean ± standard deviation (*n* = 3). Different letters (a, b, c, d, e) in the same column indicate significant differences between extracts (*p* < 0.05).

SWE Extracts	Extraction Yield (%)	TPC (mg GAE/g DW)	DPPH IC_50_ (µg/mL)	ABTS IC_50_ (µg/mL)	FRAP (µmol FSE/g)
110 °C	20.88 ± 0.78	239.53 ± 23.17 ^a^	426.88 ± 13.42	148.68 ± 13.20 ^a^	4240.38 ± 10.04 ^a^
120 °C	21.00 ± 1.49	201.75 ± 13.02 ^b^	489.49 ± 5.96	228.32 ± 16.48 ^b^	4092.98 ± 92.82 ^b^
140 °C	20.83 ± 0.62	162.52 ± 7.14 ^c^	460.21 ± 14.81	232.97 ± 4.23 ^b^	3398.98 ± 57.23 ^c^
160 °C	20.97 ± 1.74	122.31 ± 5.89 ^d^	496.15 ± 8.75	220.40 ± 7.83 ^b^	2728.06 ± 35.04 ^d^
180 °C	20.29 ± 0.93	126.19 ± 6.33 ^d^	583.47 ± 14.68	256.59 ± 5.55 ^b^	2464.32 ± 68.44 ^e^

IC_50_ = In vitro concentration required to decrease the reactivity of the studied radical species in the tested media by 50%. GAE = gallic acid equivalent; FSE = ferrous sulphate equivalent.

**Table 2:** Identification and quantification (mg/g DW) of phenolic compounds present in *C. sativa* shells SWE extracts by HPLC-PDA analysis. Results are expressed as mean ± standard deviation (*n* = 3).

Phenolic Compound	110 °C	120 °C	140 °C	160 °C	180 °C
	mg/g DW				
**Alkaloids**					
Caffeine	1.18 ± 0.06	1.14 ± 0.06	1.01 ± 0.05	0.33 ± 0.02	0.44 ± 0.02
**Chalconoids**					
Phloridzin	0.83 ± 0.04	0.50 ± 0.03	ND	ND	ND
**Flavanols**					
Catechin	4.10 ± 0.20	3.65 ± 0.18	1.60 ± 0.08	0.74 ± 0.04	0.69 ± 0.03
Epicatechin	1.68 ± 0.08	1.14 ± 0.06	0.75 ± 0.04	<LOQ	<LOQ
**Flavonols**					
Rutin	1.04 ± 0.05	0.64 ± 0.03	0.69 ± 0.03	0.103 ± 0.005	0.136 ± 0.007
Flavonones					
Naringin	<LOQ	<LOQ	ND	ND	ND
**Phenolic acids**					
3,5-di-caffeolquinic acid	<LOQ	<LOQ	<LOQ	ND	ND
4-O-caffeyolquinic acid	0.49 ± 0.02	0.46 ± 0.02	0.40 ± 0.02	0.34 ± 0.02	0.21 ± 0.01
4,5-DI-O-caffeoylquinic acid	0.126 ± 0.006	0.22 ± 0.01	0.34 ± 0.02	0.026 ± 0.001	0.28 ± 0.01
Caftaric acid	0.44 ± 0.02	0.26 ± 0.01	0.53 ± 0.03	0.16 ± 0.01	0.58 ± 0.03
Caffeic acid	0.17 ± 0.01	0.16 ± 0.01	0.12 ± 0.01	0.069 ± 0.003	0.12 ± 0.01
Clorogenic acid	0.43 ± 0.02	0.27 ± 0.01	0.18 ± 0.01	0.081 ± 0.004	0.13 ± 0.01
p-Coumaric acid	<LOD	<LOD	<LOD	<LOD	<LOD
Ellagic acid	0.136 ± 0.007	0.157 ± 0.008	0.181 ± 0.009	0.064 ± 0.003	0.108 ± 0.005
Gallic acid	5.99 ± 0.30	5.08 ± 0.25	4.64 ± 0.23	3.48 ± 0.17	2.40 ± 0.12
Ferulic acid	<LOQ	<LOQ	<LOQ	0.011 ± 0.001	0.056 ± 0.003
Neochlorogenic acid	0.71 ± 0.04	0.59 ± 0.03	0.49 ± 0.02	0.21 ± 0.01	0.28 ± 0.01
Protocatechuic acid	1.68 ± 0.08	1.37 ± 0.07	1.15 ± 0.06	1.31 ± 0.07	1.59 ± 0.08
Sinapic acid	ND	ND	ND	<LOQ	<LOQ
Syringic acid	ND	ND	ND	0.019 ± 0.001	0.063 ± 0.003
Vanillic acid	1.02 ± 0.05	0.91 ± 0.05	0.55 ± 0.03	0.084 ± 0.004	0.12 ± 0.01
Quercetin-3-O-galactoside	0.115 ± 0.006	0.099 ± 0.005	0.136 ± 0.007	0.012 ± 0.001	0.120 ± 0.006
Quercetin-3-O-glucopyranoside	0.057 ± 0.003	0.069 ± 0.003	0.081 ± 0.004	0.043 ± 0.002	0.036 ± 0.002
**Stilbenoids**					
Resveratrol	<LOD	<LOD	<LOD	ND	ND
Trans-polydatin	<LOQ	<LOD	<LOD	<LOD	<LOD
**Total**	20.20	16.71	12.85	7.07	7.37

ND, not detected; LOD, limit of detection; LOQ, limit of quantitation.

**Table 3:** Superoxide anion radical (O_2_^•**−**^), hypochlorous acid (HOCl), peroxyl radical (ROO^•^) and peroxynitrite (ONOO^-^) scavenging capacities of *C. sativa* shells SWE extracts. Values are expressed as mean ± standard deviations (*n* = 3). Different letters (a, b, c, d) in the same column indicate significant differences between extracts (*p* < 0.05).

	Reactive Oxygen Species			Reactive Nitrogen Species	
SWE Extracts	O_2_^•−^	HOCl	ROO^•^	ONOO^-^ in Presence of HCO_3_^−^	ONOO^-^ in Absence of HCO_3_^−^
	IC_50_ (µg/mL)	IC_50_ (µg/mL)	(µmol TE/mg DW)	IC_50_ (µg/mL)	
110 °C	44.36 ± 0.34 ^cd^	4.47 ± 0.29 ^b^	0.73 ± 0.024 ^b^	2.52 ± 0.19 ^c^	2.53 ± 0.22 ^bc^
120 °C	46.31 ± 1.37 ^d^	6.49 ± 0.18 ^b^	0.54 ± 0.016 ^cd^	1.41 ± 0.11 ^b^	3.32 ± 0.19 ^c^
140 °C	31.14 ± 2.05 ^b^	8.27 ± 0.77 ^b^	0.61 ± 0.037 ^bc^	2.77 ± 0.29 ^c^	2.09 ± 0.21 ^b^
160 °C	35.34 ± 3.14 ^bc^	22.85 ± 1.67 ^c^	0.50 ± 0.032 ^cd^	2.79 ±0.26 ^c^	1.89 ± 0.09 ^b^
180 °C	73.18 ±1.86 ^e^	20.74 ± 1.27 ^c^	0.49 ± 0.009 ^d^	2.93 ± 0.03 ^c^	5.94 ± 0.30 ^d^
**Positive control**					
Gallic acid	23.82 ± 0.82 ^a^	1.68 ± 0.16 ^a^	4.23 ± 0.25 ^a^	0.30 ± 0.03 ^a^	0.05 ± 0.00 ^a^

IC_50_ = In vitro concentration required to decrease the reactivity of the studied reactive species in the tested media by 50%. TE = Trolox Equivalents.

**Table 4:** Minimum Inhibition Concentration (MIC) and Minimum Bactericidal Concentration (MBC) of *C. sativa* shells SWE extracts on the bacterial strains tested, at concentrations range of 2–64 mg/mL. Values are expressed as mean (*n* = 3).

SWE Extracts		Gram-Negative			Gram-Positive		
		*E. coli* ATCC 25922	*E. coli* ATCC 8739	*E. coli* CTX M2 *	*S. aureus* ATCC 25913	*S. aureus* MRSA *	*E. faecalis*
110 °C	MIC	8	4	4	-	4	64
	MBC	64	-	-	-	32	-
120 °C	MIC	8	4	8	64	8	2
	MBC	64	-	-	-	16	-
140 °C	MIC	4	16	4	4	32	64
	MBC	-	-	-	-	64	-
160 °C	MIC	4	4	4	4	4	8
	MBC	32	-	-	64	32	64
180 °C	MIC	8	8	-	4	4	16
	MBC	16	4	-	64	16	64

* drug-resistant bacteria; - absence of MIC/MBC.

**Table 5:** IC_50_ values of oral model cellular lines treated with *C. sativa* shells SWE extracts, measured by MTT assay. Values are expressed as mean (*n* = 3). IC_50_ = In vitro concentration required to decrease cell viability by 50%.

	Cell Lines IC_50_ (µg/mL)	
SWE Extracts	HSC3	TR146
110 °C	1325.03	468.15
120 °C	1730.39	496.45
140 °C	1862.17	663.83
160 °C	1923.94	553.79
180 °C	1707.33	572.18

## Data Availability

Not applicable.

## Supplementary Materials

The following supporting information can be downloaded at: https://www.mdpi.com/article/10.3390/ijms232314956/s1.
