# Supplementary material for: New Perspectives on the Sustainable Employment of Chestnut Shells as Active Ingredient against Oral Mucositis: A First Screening
Source: Int J Mol Sci. 2022 Nov 29;23(23):14956. doi: 10.3390/ijms232314956 (PMC9737246; doi:10.3390/ijms232314956)
Supplement: Supplementary file 1 [file ijms-23-14956-s001.zip › ijms-1957716-supplementary.pdf]

Supplementary Information

Table S1. Calibration data used for the quantification of individual phenolic compounds in chestnut shells extracts.

Phenolic Compounds	(m ± Δm)[a]	(b ± Δb)[b]	r^2	LOD[c] (mg/L)	LOQ[d] (mg/L)
**Alkaloids**					
Caffeine	54054 ± 257	6136 ± 307	0.9999	0.313	1.043
**Chalconoids**					
Phloridzin	47726 ± 42	78 ± 8	0.9999	0.125	0.416
**Flavanols**					
Catechin	13826 ± 56	3021 ± 1127	0.9999	0.568	1.89
Epicatechin	15117 ± 60	1178 ± 118	0.9999	0.558	1.86
**Flavonols**					
Rutin	83207 ± 51	1937 ± 1034	0.9999	0.087	0.289
Quercetin-3-*O*-galactoside	23597 ± 27	466 ± 47	0.9999	0.159	0.531
Quercetin-3-*O*-glucopyranoside	48964 ± 119	17.0 ± 0.9	0.9999	0.161	0.535
**Flavonones**					
Naringin	36536 ± 25	1384 ± 499	0.9999	0.095	0.317
**Phenolic acids**					
3,5-di-*O*-caffeolquinic acid	62289 ± 829	-11003 ± 3153	0.9991	0.341	1.13
4-*O*-caffeyolquinic acid	12334 ± 202	-1032 ± 52	0.9995	0.19	0.632
4,5-di-*O*-caffeoylquinic acid	28503 ± 410	32 ± 3	0.9991	0.383	1.27
Caftaric acid	18700 ± 50	966 ± 48.3	0.9999	0.177	0.591
Caffeic acid	69190 ± 45	124 ± 12	0.9999	0.090	0.302
p-Coumaric acid	146520 ± 1176	-33763 ± 3376	0.9991	1.70	5.68
Ellagic acid	44947 ± 1040	-30767 ± 20152	0.9973	3.09	10.3

Gallic acid	54220 ± 97	57.7 ± 6	0.9999	0.120	0.400
trans-Ferulic acid	61448 ± 39	1501.6 ± 789	0.9999	0.089	0.298
Neochlorogenic acid	50502 ± 344	5751 ± 288	0.9998	0.449	1.495
Protocatechuic acid	32064 ± 24	1024 ± 477	0.9999	0.104	0.346
Sinapic acid	29742 ± 16	124 ± 12	0.9999	0.075	0.25
Syringic acid	61262 ± 72	2088 ± 209	0.9999	0.165	0.551
Vanillic acid	36254 ± 55	1215 ± 121	0.9999	0.215	0.715
Chlorogenic acid	27244 ± 57	-1445 ± 144	0.9999	0.292	0.972
**Stilbenoids**					
Resveratrol	181186 ± 1303	-16059 ± 803	0.9998	0.474	1.579
Trans-polydatin	64539 ± 221	4280 ± 214	0.9999	0.226	0.753

[a]m: slope ± standard deviation (n = 5) expressed in µV min/mg L; [b]b: intercept ± standard deviation (n = 5) expressed in µV min; [c]LOD: limit of detection; [d]LOQ: limit of quantification.
